# Unraveling Defect-Dependent Conductivity-Type Switching in CuFe_2_O_4_ for Enhanced Photoelectrocatalytic Reduction of Benzaldehyde

**DOI:** 10.1021/acsami.5c24965

**Published:** 2026-02-19

**Authors:** Yen-Chun Huang, Manoj Kumar Mohanta, Jun-Lin Fong, Abdul M. Reyes, Sebastian E. Reyes-Lillo, Chang-Ming Jiang

**Affiliations:** † Department of Chemistry, 33561 National Taiwan University, 10617 Taipei, Taiwan; ‡ Center for Emerging Materials and Advanced Devices, National Taiwan University, 10617 Taipei, Taiwan; § Departamento de Física y Astronomía, Facultad de Ciencias Exactas, 28087 Universidad Andres Bello, 837-0136 Santiago, Chile; ∥ SPN Tumaco, Universidad Nacional de Colombia, Kilómetro 30-31, Vía Nacional Tumaco 525560, Colombia

**Keywords:** copper ferrite, spinel, photoelectrochemistry, photocathode, oxygen vacancy, benzaldehyde reduction, point defects

## Abstract

Photoelectrocatalytic (PEC) reduction provides a sustainable route for upgrading biomass-derived feedstocks with reduced energy requirements, yet remains largely unexplored beyond hydrogen evolution and CO_2_ reduction due to the scarcity of stable photocathodes. Here, we report a defect-engineered CuFe_2_O_4_ photocathode that enables directly quantified PEC reduction of benzaldehyde to benzyl alcohol using a single-component, earth-abundant oxide. By controlling annealing temperature and oxygen partial pressure, CuFe_2_O_4_ is systematically tuned from n-type to p-type conductivity. Electrochemical measurements, X-ray and ultraviolet photoelectron spectroscopy, and first-principles defect calculations collectively show that oxygen-rich annealing conditions suppress deep donor-type oxygen vacancies while stabilizing shallow acceptor-type copper vacancies, resulting in enhanced hole concentration and improved charge transport. In a mixed acetonitrile/water electrolyte employing 1,4-benzoquinone as a redox mediator, the optimized CuFe_2_O_4_ photocathode achieves stable photoelectrochemical operation over 18 h under continuous illumination with a benzyl alcohol production rate of 2.57 μmol/h at −0.50 V vs Ag/AgNO_3_, corresponding to a Faradaic efficiency of 51.3%. This PEC approach lowers the required applied potential by ∼1 V compared to traditional electrocatalytic methods, offering a more energy-efficient route for carbonyl reduction. These findings establish CuFe_2_O_4_ as a viable photocathode platform for sustainable photoelectrocatalytic organic transformations under mild reaction conditions.

## Graphical abstract

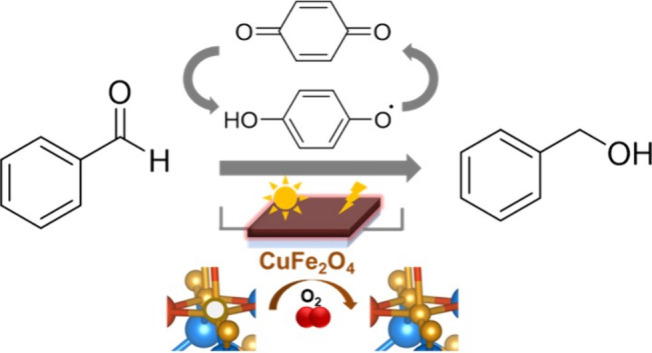

## Introduction

Photoelectrocatalysis (PEC) provides a sustainable platform for converting solar energy into chemical fuels and value-added products. While PEC water splitting has been extensively investigated,^1,2^ the sluggish kinetics of the oxygen evolution reaction (OER) have motivated the development of alternative oxidation pathways that utilize biomass-derived substrates, yielding high-value chemicals while simultaneously producing green hydrogen.^3,4^ Recent examples include PEC glucose oxidation using Pt-decorated TiO_2_, producing glucaric acid,^5^ and selective BiVO_4_-driven oxidation, such as the conversion of benzyl alcohol to benzaldehyde,^6,7^ glycerol to dihydroxyacetone, and 5-hydroxymethylfurfural to 2,5-furandicarboxylic acid.^8,9^ These advancements highlight PEC as an energy-efficient approach for upgrading biomass feedstocks into renewable fuels and chemical precursors.^10^

While PEC oxidation of organic compounds has been well-documented, including the activation of C–H and C–C bonds in nonaqueous environments,^6,11,12^ PEC reduction reactions remain significantly less explored. Electrocatalytic studies have shown that benzaldehyde and furfural can be reduced under ambient conditions without external hydrogen sources,^13,14^ but photocathode-driven reductions are rare. Beyond hydrogen evolution reaction (HER) and CO_2_ reduction,^15,16^ only a few PEC reductive transformations—such as nitrobenzene-to-aniline conversion,^17^ H_2_O_2_ production,^18^ and ammonia synthesis^19,20^ —have been reported. To date, the only reported instance of PEC carbonyl reduction employs Au-cluster-sensitized α-Bi_2_O_3_ (*E*_*g*_ = 2.80 eV),^21^ where noble-metal clusters function as both light absorbers and catalytic sites, enabling rapid benzaldehyde consumption under dilute (0.1 mM) substrate conditions but without direct product identification or Faradaic efficiency determination. Thus, there is a clear need for earth-abundant photocathodes that can drive carbonyl reduction under practical concentrations and quantifiable efficiencies.

Photocathodes are typically based on p-type semiconductors, where downward band-bending at the solid-electrolyte interface promotes electron transfer to solution-phase reactants. Metal oxides are attractive for this purpose due to their low fabrication costs and tunable bandgaps; however, most exhibit intrinsic n-type conductivity^22^ arising from the low formation energy of oxygen vacancies. A group of Cu-containing oxides, including Cu_2_O, CuFeO_2_, CuBi_2_O_4_, and CuNb_3_O_8_, exhibits inherent p-type behavior and favorable conduction band positions, making them promising candidates for photocathodes.^23,24^ However, instability under cathodic bias, short carrier diffusion lengths, and detrimental surface states continue to limit their performance.^25,26^ As a result, efficient and stable metal-oxide photocathodes for PEC organic reduction remain scarce.

CuFe_2_O_4_ is an earth-abundant inverse spinel oxide with a band gap of ∼1.54–1.95 eV and a conduction band sufficiently negative for HER.^27–29^ Nonetheless, its reported cathodic photocurrent densities rarely exceed 0.5 mA/cm^2^, likely due to poor charge carrier mobility, insufficient crystallinity, or suboptimal electrical contact with the fluorine-doped tin oxide (FTO) substrate. Interestingly, Liu et al. synthesized CuFe_2_O_4_ films via chemical bath deposition, achieving a water-oxidizing photocurrent density of 0.5 mA/cm^2^ at 1.6 V vs RHE in 1 M NaOH under simulated solar illumination.^30^ Einert et al. prepared mesoporous n-type CuFe_2_O_4_ films, reporting an anodic photocurrent density of 0.18 mA/cm^2^ for sulfite oxidation.^31^ These diverse studies, employing different synthesis methods and film morphologies, underscore the versatility of CuFe_2_O_4_. However, a systematic understanding of how defect populations govern the conductivity-type switching and how such control impacts PEC reductive performance remains lacking.

Herein, we address this knowledge gap by demonstrating that defect-engineering enables CuFe_2_O_4_—an earth-abundant inverse spinel oxide—to function as an efficient and stable photocathode for photoelectrocatalytic carbonyl reduction. By systematically tuning annealing temperature and oxygen partial pressure, we control the relative populations of oxygen-vacancies (V_O_) and Cu-vacancies (V_Cu_), including a transition from n-type to p-type conductivity. A combination of electrochemical measurements, XPS/UPS analysis, and first-principles defect calculations reveals that synthesis in an oxygen-rich environment suppresses deep donor-type V_O_ defects while stabilizing shallow acceptor-type V_Cu_, thereby enhancing hole concentration and charge-transport properties.

Using this optimized CuFe_2_O_4_ photocathode in conjunction with a molecular redox mediator (1,4-benzoquinone), we achieve sustained photoelectrochemical reduction of benzaldehyde to benzyl alcohol under simulated solar illumination. Unlike the previously reported Au-cluster-sensitized α-Bi_2_O_3_ system, the present approach employs a single-component, noble-metal-free oxide photocathode and enables direct product identification and Faradaic efficiency quantification at substantially higher substrate concentrations. These findings establish defect-engineered CuFe_2_O_4_ as a viable platform for PEC carbonyl reduction and highlight defect control coupled with redox mediation as a general strategy for advancing photoelectrocatalytic organic reductions.

## Experimental Section

### Synthesis of CuFe_2_O_4_ Films

CuFe_2_O_4_ films were deposited on fluorine-doped tin oxide (FTO) coated glass substrate using a previously established sol–gel method.^28^ To prepare the precursor solution, 0.2 M copper­(II) nitrate hydrate (>99.99%, Sigma-Aldrich), 0.2 M iron­(III) nitrate nonahydrate (≥99.95%, Sigma-Aldrich), and 0.4 M citric acid (99%, Sigma-Aldrich) were dissolved in absolute ethanol (>99.8%%, Riedel-de Häen). After sonication for 20 min, 0.25 M ethylene glycol (99%, Sigma-Aldrich) was added, and the mixture was stirred overnight before being filtered through a 0.2 μm PTFE syringe filter.

The FTO substrates were cleaned sequentially in deionized water, isopropyl alcohol, and acetone in an ultrasonic bath for 10 min each, followed by nitrogen blow-dry and a 10 min UV-ozone treatment. As shown in Figure S1, 200 μL of the filtered precursor solution was dispensed onto a 2.5 × 2.5 cm^2^ FTO substrate and spin-coated at 3000 rpm for 60 s, with an acceleration rate of 600 rpm/s. The films were heated on a hot plate at 120 °C for 10 min and then in a box furnace (CWF 1100, Carbolite Gero) at 450 °C for 1 h in the air. The spin-coating and the pyrolysis steps were repeated four times to achieve the desired film thickness. For the final annealing step, the films were either placed in the same box furnace at 600–750 °C for 20 min or in a tube furnace (TS1-1200, Carbolite Gero) under a controlled nitrogen/oxygen atmosphere at 700–750 °C for 20 min, with a ramp-up rate of 5 °C/min.

### Photoelectrochemical Measurements in an Aqueous Environment

Electrochemical measurements were performed in a three-electrode setup using a custom-built undivided cell with a Metrohm PGSTAT204 potentiostat. A 1.25 × 1.25 cm^2^ CuFe_2_O_4_ film on FTO served as the working electrode, while a platinum wire was the counter electrode. For aqueous measurements, an Ag/AgCl reference electrode immersed in saturated KCl solution was used. The electrolyte was 1 M NaOH (99%, UniRegion Bio-Tech), with either 0.2 M Na_2_S_2_O_8_ (≥99%, Honeywell) as an electron scavenger or 0.2 M Na_2_SO_3_ (98–100%, Sigma-Aldrich) as a hole scavenger. Illumination was provided by an AM 1.5G LED solar simulator (LSH-7320, Oriel) at 1 sun intensity (100 mW/cm^2^). Linear sweep voltammetry (LSV) scans were conducted at a scan rate of 10 mV/s, and potentials were referenced to the reversible hydrogen electrode (RHE) using the equation:

$$E_{RHE}=E_{Ag/AgCl}+0.207\;V+\left(0.0592\;V\times \mathrm{pH}\right)$$

Mott–Schottky analysis was conducted under illumination using a Zennium Pro potentiostat (Zahner-Elektrik) with a 40 mV voltage perturbation amplitude at 1 kHz frequency. Photoelectrochemical impedance spectroscopy (PEIS) was conducted in the dark and under illumination over a frequency range of 100 mHz to 1 MHz. Frequency-dependent impedance data were fitted using Zahner Analysis software.

### Photo-Driven Reduction of Benzaldehyde

Photoelectrocatalytic reduction of benzaldehyde was performed in a 4:1 (v/v) acetonitrile/water solution using an Ag/AgNO_3_ reference electrode. A 1.25 × 1.25 cm^2^ CuFe_2_O_4_ film was immersed in a 20 mL solution containing 0.4 mmol benzaldehyde (≥99%, Sigma-Aldrich) and 0.4 mmol 1,4-benzoquinone (≥98%, Sigma-Aldrich). Each constant-potential photoelectrocatalysis experiment was illuminated by AM 1.5G simulated solar light at 100 mW/cm^2^ intensity and continued for 18 h without interruption.

Reaction products were analyzed using a Shimadzu GC-2010 gas chromatography (GC) system equipped with a Trajan BP1 fused silica capillary column (60 m × 0.53 mm ID × 3.0 μm). A 1 μL electrolyte sample was injected via autosampler, with nitrogen as the carrier gas at a 3.0 mL/min flow rate. A flame ionization detector (FID) detected current changes and generated a gas chromatogram. The column temperature was initially set at 60 °C for 1 min, then ramped to 100 °C at 10.0 °C/min, and held for 10 min. It was then increased to 150 °C at 20.0 °C/min and held for 3 min, followed by a final ramp to 250 °C at 40 °C/min, held for 3 min.

## Results and Discussion

### Crystallographic and Morphological Characterization

CuFe_2_O_4_ crystallizes in an inverse spinel structure with tetragonal symmetry (space group *I*4_1_/*amd*) and lattice constants of *a* = 5.80 Å and *c* = 8.73 Å. Cu and Fe ions are randomly distributed across the 16*d* Wyckoff positions, while Fe and O ions occupy the 8*a* and 32*e* positions, respectively. CuFe_2_O_4_ films were synthesized on FTO glass substrates via spin-coating and annealed at varying temperatures and atmospheres; for example, CFO600-O20 denotes annealing at 600 °C in air (∼20% oxygen), while CFO750-O50 refers to annealing at 750 °C in a 1:1 N_2_/O_2_ atmosphere.

Figure 1a confirms phase-pure tetragonal CuFe_2_O_4_ across all samples, with diffraction peaks at 30.3°, 36.1°, 37.3°, and 44.0° indexed to the (112), (211), (202), and (220) planes.^32^ Increasing the annealing temperature (*T*_*a*_) from 600 to 750 °C yields sharper and more intense reflections, most notably the (211) peak, signifying improved crystallinity. Plane-view SEM images (Figure S2) reveal that the average CuFe_2_O_4_ grain size increased from ∼15 nm to ∼100 nm over this temperature range. Although bulk CuFe_2_O_4_ typically crystallizes at 800–1000 °C,^28^ such high temperatures exceed the ∼560 °C glass transition temperature of FTO; thus, the 650–750 °C window employed here represents a practical compromise between crystallization and substrate stability. Cross-sectional SEM (Figure S3) reveals a uniform film thickness of ∼220 nm.

**Figure 1:**
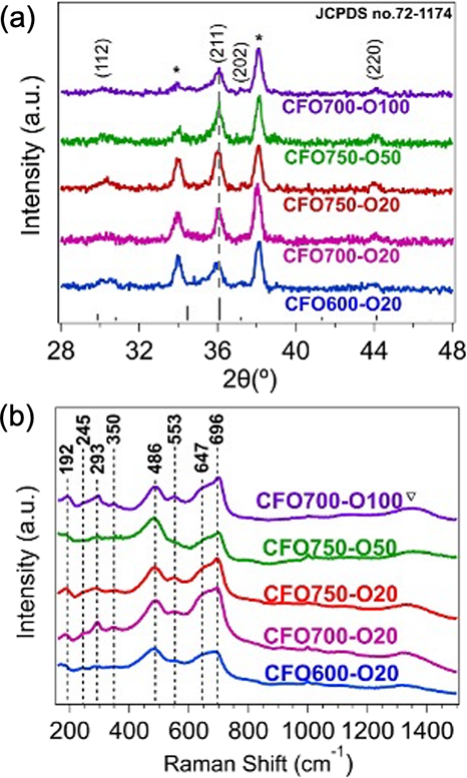
(a) Grazing incidence X-ray diffraction patterns and (b) Raman spectra of CuFe_2_O_4_ films synthesized with different annealing conditions. The asterisks indicate the diffraction peaks of SnO_2_ in the FTO glass substrates, while the inverse triangle indicates the Raman peak of α-Fe_2_O_3_. The reference diffraction pattern is calculated according to the JCPDS card No. 72-1174.

Raman spectra (Figure 1b) display characteristic peaks at 192, 245, 293, 350, 486, 553, 647, and 696 cm^–1^, which can all be attributed to the T_2g_, E_g_, or A_1g_ vibrational modes of CuFe_2_O_4_.^33,34^ A weak band at 1330 cm^–1^ suggests the presence of α-Fe_2_O_3_,^35^ although the absence of corresponding GIXRD features indicates only short-range ordering of this impurity phase.

### Photoelectrochemical Performance and Conductivity Switching

The semiconductor character of CuFe_2_O_4_ can be fine-tuned from n-type to p-type by controlling *T*_*a*_. In linear scan voltammetry (LSV), anodic currents are defined as positive, and cathodic currents are defined as negative. With Na_2_SO_3_ as a hole scavenger, CFO600-O20 exhibits an anodic photocurrent onset at 0.95 V vs RHE and reaches a photocurrent density of 0.09 mA/cm^2^ at 1.23 V vs RHE under 1 sun illumination (Figure 2a), confirming n-type behavior that allows photogenerated holes to be extracted at the CuFe_2_O_4_-electrolyte interface due to upward band-bending. The α-Fe_2_O_3_ impurity is not expected to contribute significantly to the anodic photocurrent for two reasons: first, the impurity phase was minor in CuFe_2_O_4_ films and lacked high crystallinity. Second, back-side illumination led to an ∼100% enhancement in photocurrent compared to front-side illumination (Figure S4), suggesting that electron mobility is lower than hole mobility in CuFe_2_O_4_, in contrast to the hole diffusion limitation of α-Fe_2_O_3_.^36^

**Figure 2:**
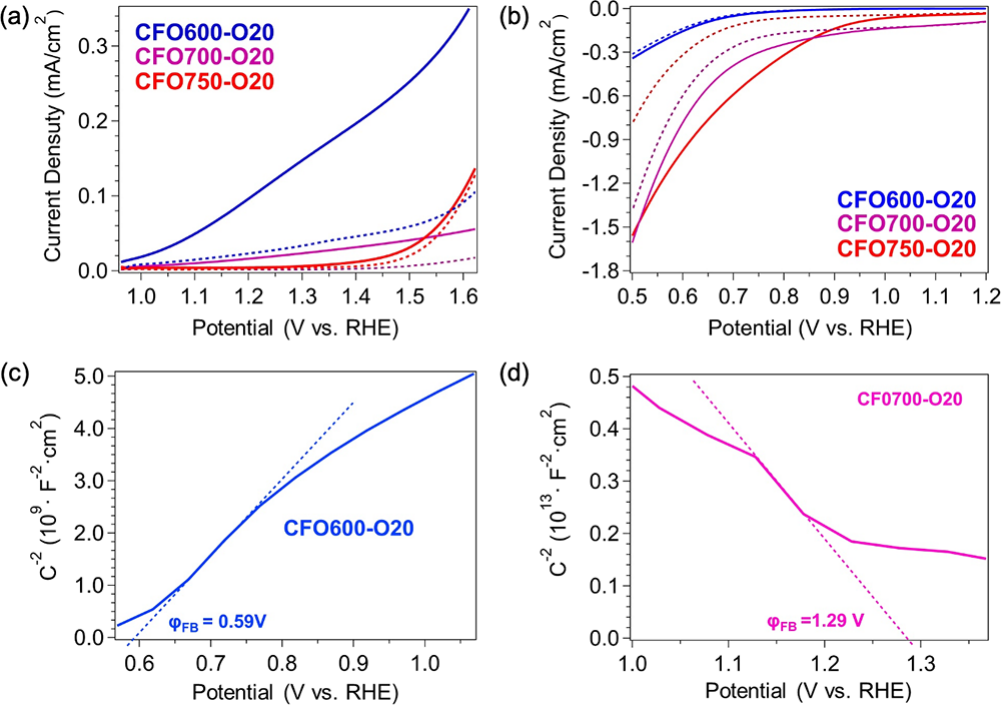
(a-b) Linear sweep voltammetry (LSV) plots measured from CFO600-O20, CFO700-O20, and CFO750-O20 in (a) 1 M NaOH with 0.2 M Na_2_SO_3_ and (b) 1 M NaOH with 0.2 M Na_2_S_2_O_8_. The dashed lines were acquired in dark conditions, while the solid lines were recorded under back-side illumination of AM 1.5G simulated solar light at 1 sun intensity. (c-d) Mott–Schottky plots measured under back-side AM 1.5G illumination from (c) CFO600-O20 in 1 M NaOH with 0.2 M Na_2_SO_3_ and (d) CFO700-O20 in 1 M NaOH with 0.2 M Na_2_S_2_O_8_.

As *T*_*a*_ increases, the anodic response diminishes: CFO700-O20 reaches only 0.034 mA/cm^2^ at 1.23 V vs RHE, and CFO750-O20 shows negligible anodic photocurrent. When Na_2_S_2_O_8_ is used as an electron scavenger (Figure 2b), CFO600-O20 becomes nearly inactive, while CFO700-O20 and CFO750-O20 deliver cathodic photocurrents of −0.22 and −0.74 mA/cm^2^ at 0.5 V vs RHE, respectively. These trends clearly illustrate a transition from n-type to p-type conduction as *T*_*a*_ increases from 600 to 750 °C.

Mott–Schottky analysis corroborates these findings. CFO600-O20 shows a positive slope with a flat band potential (φ_FB_) of 0.59 V vs RHE (Figure 2c), consistent with n-type character. CFO700-O20 and CFO750-O20 exhibit negative slopes (Figure 2d and 3b), indicating p-type behavior. The smaller magnitude of the slope for CFO750-O20 implies a higher majority carrier concentration. Corresponding φ_FB_ values shift to 1.29 V (CFO700-O20) and 1.31 V (CFO750-O20) vs RHE.

**Figure 3:**
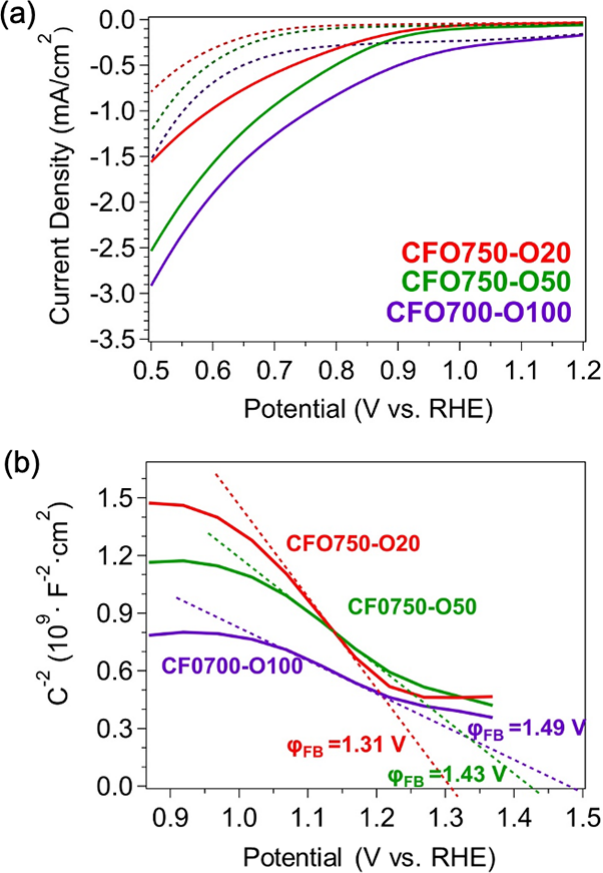
(a) LSV plots and (b) Mott–Schottky plots measured from CFO750-O20, CFO750-O50, and CFO700-O100 in 1 M NaOH and 0.2 M Na_2_S_2_O_8_ added as a sacrificial electron acceptor.

### Effect of Defects and Oxygen Partial Pressure on Conductivity

The observed conductivity-type switching arises from competition between two intrinsic point defects in CuFe_2_O_4_: oxygen vacancies (V_O_), which act as n-type donors, and copper vacancies (V_Cu_), which act as p-type acceptors. Because Cu-containing oxides often favor V_Cu_ formation,^37^ higher annealing temperatures can increase V_Cu_ concentrations sufficiently to compensate V_O_, shifting CuFe_2_O_4_ toward p-type behavior. Prior work on CuO photocathodes similarly showed enhanced p-type conductivity after annealing in oxygen-rich environments.^37^

To further probe this effect, CuFe_2_O_4_ films were annealed in oxygen-rich atmospheres (1:1 N_2_/O_2_ or pure O_2_). Annealing at 750 °C in pure O_2_ degraded the FTO substrate, as evidenced by diminished SnO_2_ reflections at 34° and 38° (Figure S5a), indicating that the FTO layer could not withstand such conditions.^38^ The disintegration of the FTO back contact resulted in decreased photocurrent density compared to CFO750-O20 (Figure S5b). Therefore, we adopted 700 °C for synthesizing CuFe_2_O_4_ photocathodes in pure O_2_ to preserve substrate integrity.

Figure 3a shows that cathodic photocurrent density increases systematically with oxygen content in the annealing atmosphere, rising from −0.74 mA/cm^2^ for CFO750-O20 to −1.3 mA/cm^2^ for CFO750-O50 and further to −1.5 mA/cm^2^ for CFO700-O100 at 0.5 V vs RHE. Concurrently, the photocurrent onset shifts from 0.94 to 1.17 V RHE, while anodic photocurrents remain negligible even in the presence of Na_2_SO_3_ (Figure S6). These changes reflect suppression of V_O_ donor states following annealing in an oxygen-rich environment.

Mott–Schottky analysis shows a reduction in slope as the oxygen partial pressure in the annealing atmosphere increases (Figure 3b), consistent with higher hole concentrations. The flat band potential also shifted to more positive values, further supporting the enhanced p-type character in CuFe_2_O_4_. To ensure that the conductivity assignments were not dependent on a single modulation frequency, measurements were performed at 100–5000 Hz for all CuFe_2_O_4_ samples (Figure S7). Although slight shifts in the x-intercepts are observed due to nonideal semiconductor–electrolyte interfaces, the extracted flat band potentials follow a consistent trend across all frequencies.

### Defect Formation Energies Calculations

To support our experimental findings, we performed first-principles calculations to evaluate the formation energies and electronic levels of V_O_ and V_Cu_ in CuFe_2_O_4_. Several cation-disordered configurations were explored using a 52-atom unit cell containing eight formula units (Figure S8), and multiple magnetic arrangements were tested (Table S3). Structural optimizations of the unit cell volume and atomic positions were carried out using DFT+U until the Hellmann–Feynman forces were minimized to below 0.01 eV/Å. The resulting lowest-energy structure displayed antiferromagnetic spin ordering of Fe ions and was subsequently used for defect formation energies (Figure 4a) and defect transition levels (Figure 4b). We note that the calculations presented here are not intended to quantitatively predict experimental defect concentrations or carrier densities, but rather to provide mechanistic insight into how oxygen and copper vacancies influence electronic structure and conductivity trends.

**Figure 4:**
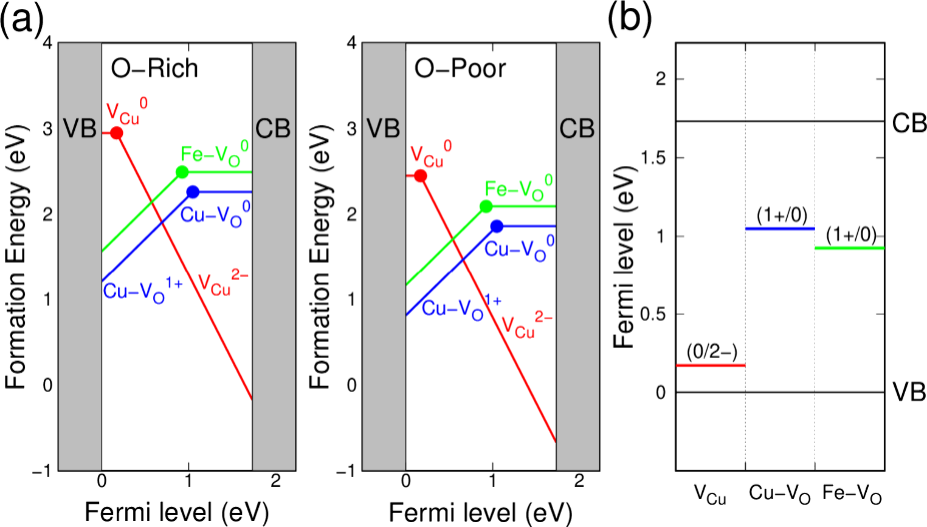
(a) Formation energies for neutral and charged Cu and O vacancies as functions of the Fermi level under oxygen-rich (O-rich) and oxygen-poor (O-poor) conditions. CB and VB denote the conduction band and valence band, respectively. Two possible positions for the oxygen vacancy adjacent to Cu and Fe ions are denoted as Cu–V_O_ and Fe–V_O_, respectively. (b) Transition levels for the Cu and O vacancy defects. The energy difference between CB and VB corresponds to the computed bandgap of 1.73 eV.

The computed defect energetics reveal transition levels near both the valence and conduction band edges under oxygen-rich and oxygen-poor conditions. Specifically, V_Cu_ acts as a shallow double acceptor with a thermodynamic transition level only 0.17 eV above the valence band maximum. Such a small ionization energy implies that V_Cu_ readily supplies free holes at room temperature, thereby promoting p-type conductivity whenever V_Cu_ formation is thermodynamically favored. In contrast, V_O_ behaves as a deep donor defect, with transition levels located 0.66–0.72 eV below the conduction band minimum. These deep levels are less likely to thermally ionize and instead serve as carrier-trapping centers that promote nonradiative recombination. The calculations also show that V_O_ formation is more favorable near Cu sites than Fe sites.

These theoretical insights provide a mechanistic explanation for the experimentally observed conductivity-type switching. Under oxygen-poor or low-temperature annealing conditions, the lower formation energy of V_O_ leads to donor-like behavior and n-type responses, as evidenced in the LSV scan (Figure 2a) and the positive-slope Mott–Schottky plot of CFO600-O20 (Figure 2c). Conversely, increasing the annealing temperature or oxygen partial pressure suppresses V_O_ while creating more V_Cu_, shifting the Fermi level toward the valence band and yielding the p-type responses observed in CFO750-O20, CFO750-O50, and CFO700-O100. This trend is corroborated by the XPS O 1*s* spectra, which show a monotonic decrease in the V_O_-related component, and by UPS measurements, where the Fermi level progressively approaches the valence band edge for the samples annealed under oxygen-rich atmospheres (vide infra).

### Chemical State Analysis via XPS

X-ray photoelectron spectroscopy (XPS) was used to investigate the chemical environments and oxygen vacancy behavior in the near-surface region of CuFe_2_O_4_ photocathodes (Figure 5). The O 1*s* XPS spectra contain three distinct components: lattice oxygen, vacancy-associated hydroxide, and adsorbed H_2_O, ordered by increasing binding energy.^39,40^ The vacancy-related signal decreases systematically with increasing *T*_*a*_ and oxygen partial pressure, while the lattice oxygen component exhibits an opposite trend. For instance, lattice oxygen increases from 54.7% in CFO600-O20 to 59.7% in CFO750-O20, with a corresponding reduction of the oxygen vacancy component from 35.6% to 30.0%. Further increases in oxygen content during annealing reduce the oxygen vacancy contribution to 25.6% (CFO750-O50) and 20.6% (CFO700-O100). Given the surface sensitivity of XPS, these measurements are used to track relative trends in defects rather than to quantify absolute bulk defect densities. These results reflect the higher formation energy of V_O_ under oxygen-rich conditions (Figure 4a) and correlate with the enhanced hole concentration and the improved cathodic photocurrent in CFO700-O100 (Figure 3).

**Figure 5:**
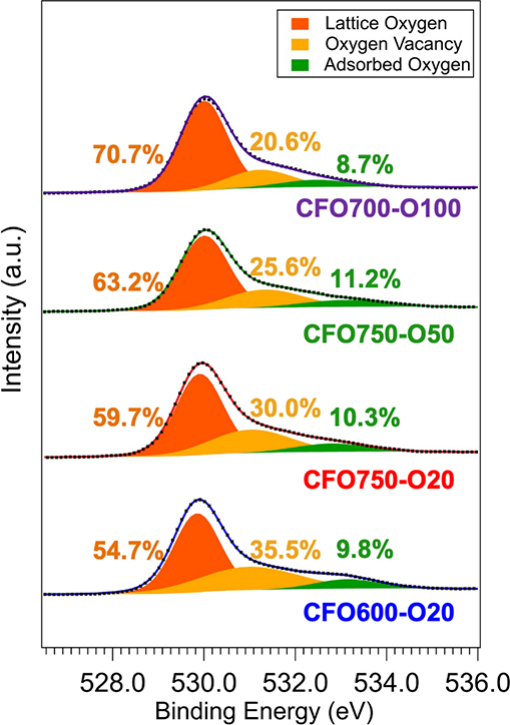
High-resolution O 1*s* X-ray photoemission spectra of CF0600-O20, CF0750-O20, CFO750-O50, and CFO700-O100. Experimental results are shown as dots, while the fitted envelopes are represented as solid lines. The lattice oxygen, oxygen vacancy, and the adsorbed H_2_O components are shown as red, orange, and green Voigt peaks, respectively.

Fe 2*p* XPS spectra show no significant differences across annealing samples (Figure S9). In the Cu 2*p* region (Figure S10), 2*p*_3/2_ and 2*p*_1/2_ peaks at ∼934.0 and ∼953.8 eV binding energies, together with pronounced shakeup satellites, confirm Cu^2+^ as the dominant oxidation state.^41^ A minor Cu^+^ contribution is also detected, particularly for CFO600-O20, potentially due to incomplete crystallization at lower annealing temperatures.^42^ The Cu LMM Auger spectra exhibit a peak at 917.4 eV kinetic energy (Figure S11), ruling out the presence of metallic Cu impurities in the near-surface region of all CuFe_2_O_4_ films.

### Energy Band Diagrams from UPS and Absorption Spectroscopy

The optical bandgaps of CuFe_2_O_4_ films, determined from Tauc analysis, range from 1.65–1.73 eV (Figure S12), consistent with our DFT+U calculated bandgap (1.73 eV) and within the reported literature range (1.54–1.80 eV).^28^ Ultraviolet photoelectron spectroscopy (UPS) was employed to determine the work functions and the valence band maxima positions (Figure 6a and 6b). While the work functions varied by less than 0.15 eV across all samples, CFO600-O20 exhibited the largest separation (1.05 eV) between the Fermi level and the valence band edge, consistent with its pronounced n-type character. In contrast, p-type samples exhibit Fermi levels closer to the valence band, with the separation decreasing progressively as oxygen content in the annealing atmosphere increases. This shift reflects V_O_ passivation and higher hole concentrations.

**Figure 6:**
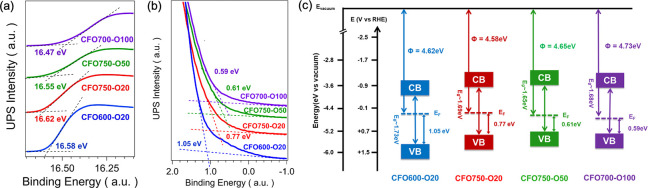
(a) Work function cutoff and (b) Fermi edge spectra acquired by ultraviolet photoelectron spectroscopy from CuFe_2_O_4_ films using He–I radiation (21.2 eV). (c) Comparison of the energy band diagrams of CuFe_2_O_4_ films presented in this work.

Using the optical bandgaps, work functions, and energy differences between Fermi levels and the valence band edges, band diagrams for CFO600-O20, CFO750-O20, CFO750-O50, and CFO700-O100 are constructed (Figure 6c), which align well with the different conductivity types observed in the LSV measurements.

### Enhancing Operational Stability of CuFe_2_O_4_ Photocathodes

Before assessing CuFe_2_O_4_ photocathodes for photodriven carbonyl reduction, we first evaluated their operational stability. In 1 M NaOH with 0.2 M Na_2_S_2_O_8_ at 0.5 V vs RHE (Figure S13), all photocathodes tested (CFO750-O20, CFO750-O50, and CFO700-O100) exhibited an initial increase in photocurrent during the first 2 min, followed by ∼40% decay over the next 30 min. This behavior is likely due to photocorrosion, a common issue in oxide-based photocathodes.^28,43–46^ This result highlights the challenges of conducting PEC reduction reactions in aqueous environments, where photogenerated electrons accumulate at the semiconductor-electrolyte interface and lead to self-reduction of the photocathode.

To enhance the stability of CuFe_2_O_4_ photocathodes, we employed two strategies. First, we replaced the aqueous electrolyte with a 4:1 (v/v) acetonitrile/water mixture containing 0.1 M LiClO_4_ as the electrolyte. This solvent system, inspired by previous studies on BiVO_4_ photoanodes using acetonitrile,^6^ aims to mitigate photocorrosion without requiring protective layers or cocatalysts. The 20% water content served as a proton source for benzaldehyde reduction. Second, we introduced 1,4-benzoquinone as a redox mediator, which is reduced by photogenerated electrons to semiquinone radicals on the CuFe_2_O_4_ surface (Figure 7a). Two semiquinone radicals subsequently reduce benzaldehyde to benzyl alcohol through a two-electron, two-proton mechanism. The utilization of a redox mediator helps maintain the applied potential within the photoelectrochemical stability window of the electrode, similar to what has been demonstrated in Li–O_2_ batteries, fuel cells, and PEC H_2_O_2_ production systems.^47–50^

**Figure 7:**
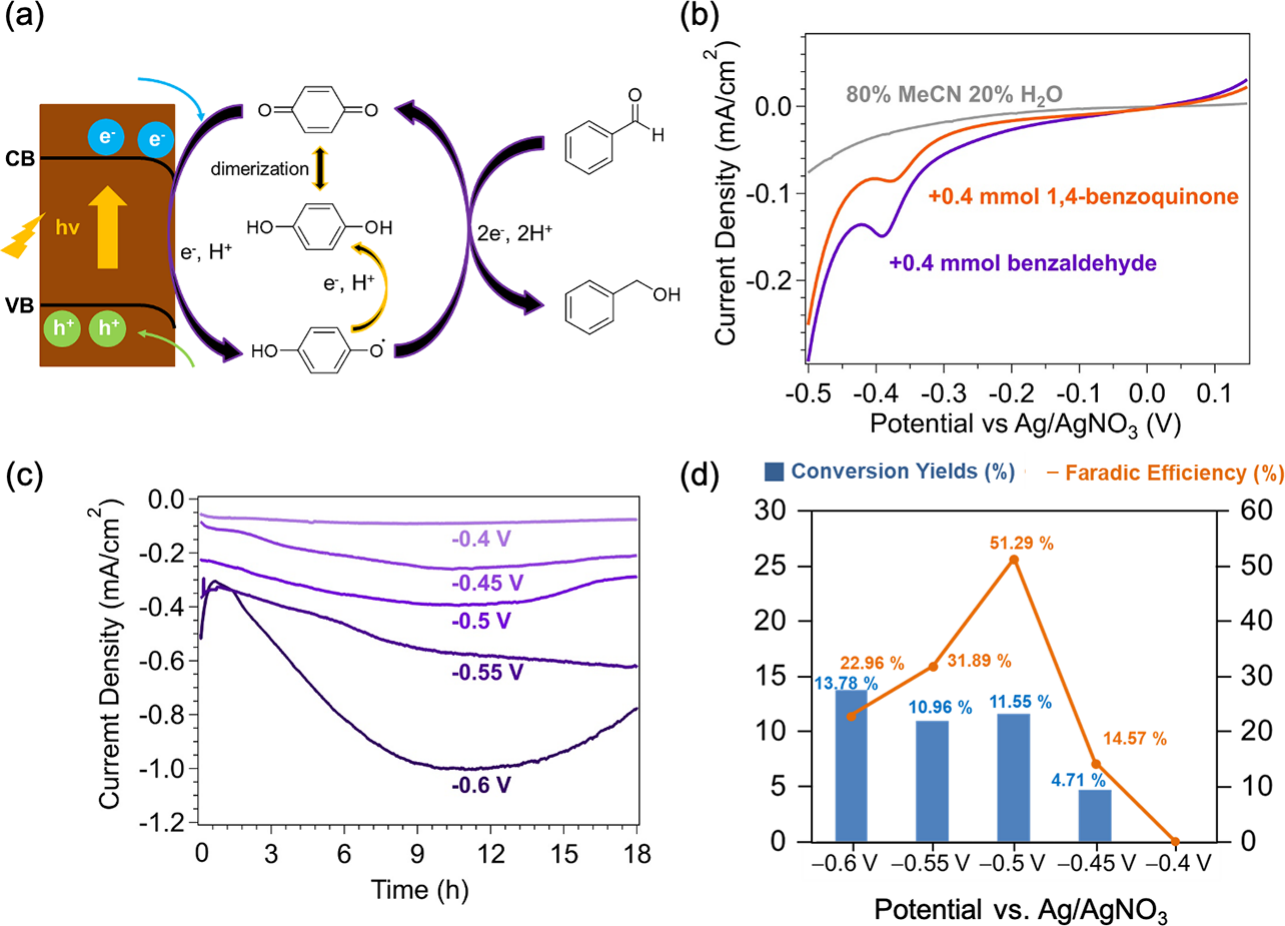
(a) Proposed mechanism of photoelectrocatalytic benzaldehyde reduction reaction mediated by 1,4-benzoquinone. (b) LSV plots measured from CFO700-100 photocathodes under AM 1.5G illumination in a 4:1 (v/v) acetonitrile/water mixture containing 0.1 M LiClO_4_ (gray), and the subsequent additions of 0.4 mmol of 1,4-benzoquinone (orange) and 0.4 mmol of benzaldehyde (purple). (c) Chronoamperometry curves measured at different applied potentials (vs Ag/AgNO_3_ reference electrode) using a CFO700-O100 photocathode for PEC benzaldehyde reduction under back-side AM 1.5G illumination at 100 mW/cm^2^ intensity. (d) Benzyl alcohol conversion yields and Faradaic efficiencies following 18 h of photoelectrocatalysis at different applied potentials.

Figure 7b shows a distinct reduction peak at −0.37 V vs Ag/AgNO_3_ upon addition of 0.4 mmol benzoquinone into 20 mL of mixed acetonitrile/water electrolyte solution. After further addition of 0.4 mmol benzaldehyde, the enhancement in cathodic photocurrent density indicates effective electron transfer from semiquinone radicals to benzaldehyde. Chronoamperometry of CFO700-O100 at applied potentials between −0.40 V to −0.60 V vs Ag/AgNO_3_ under AM 1.5G illumination (Figure 7c) revealed stable photocurrents over 18 h, except at the most negative potential. Postreaction SEM, EDX, and XPS analyses (Figures S15–S17) reveal mild surface reconstruction accompanied by partial reduction of surface Cu^2+^ to Cu^0^. Such near-surface chemical evolution is consistent with operation under prolonged cathodic bias and may contribute to the small current rise observed over time, but does not indicate bulk degradation. Importantly, GIXRD analysis after 18 h of operation at −0.50 V vs Ag/AgNO_3_ showed no noticeable decrease in CuFe_2_O_4_ diffraction peak intensities (Figure S14), confirming preservation of the bulk crystal structure during extended operation. While near-surface chemical states may evolve under prolonged cathodic bias, no evidence of bulk phase transformation or reversal of conductivity type is observed within the operational window investigated.

### Analysis of Benzyl Alcohol Production Rates and Faradaic Efficiencies

Figure 7d summarizes the benzyl alcohol conversion yields and Faradaic efficiencies obtained after 18 h of photoelectrocatalysis at different applied potentials. Gas chromatography identified benzyl alcohol as the primary product, with no detectable hydrobenzoin, the ketyl radical dimerization product.^14^ At −0.40 V vs Ag/AgNO_3_, no benzyl alcohol was detected, consistent with the low steady-state photocurrent density (<0.1 mA/cm^2^) observed under these conditions. As the applied potential became more cathodic, the conversion yield gradually increased, reaching 13.78% at −0.60 V vs Ag/AgNO_3_. The highest FE of 51.3% occurred at −0.50 V vs Ag/AgNO_3_ and declined at more cathodic potentials (31.9% and 23.0% at −0.55 V and −0.60 V, respectively), indicating the increased contribution of competing charge-consuming pathways at higher driving forces.

Control experiments highlight the essential roles of both illumination and redox mediation. Without 1,4-benzoquinone, the photocurrent collapsed to zero within 15 min at −0.50 V vs Ag/AgNO_3_ due to rapid photocorrosion (Figure S18a). In the absence of illumination, no benzyl alcohol was detected after 18 h of electrocatalysis (Figure S18b).

The nonunity FE for benzyl alcohol production reflects the presence of competing reaction pathways. One likely contribution is the hydrogen evolution reaction (HER), which is thermodynamically accessible on CuFe_2_O_4_ photocathodes^27,28^ and may increasingly compete with benzaldehyde reduction at more cathodic potentials, even in a solvent system containing only 20% water. In addition, partial further reduction of the semiquinone radical to hydroquinone, followed by quinhydrone formation with 1,4-benzoquinone, constitutes a mediator-related loss pathway (Figure 7a).^51^ This process is evidenced by the characteristic brown coloration of the electrolyte and the emergence of a UV–vis absorption band at 420 nm after prolonged operation (Figure S19); therefore, this side reaction could consume redox mediator and reduce the FE for benzyl alcohol production. Although hydroquinone may in principle be reoxidized electrochemically or via comproportionation with 1,4-benzoquinone, these regeneration pathways are not optimized under the present conditions, resulting in partial mediator loss over prolonged operation.

### Charge Dynamics Studies via Photoelectrochemical Impedance Spectroscopy

Lastly, photoelectrochemical impedance spectroscopy (PEIS) was used to investigate the electron dynamics in CFO750-O20 and CFO700-O100 at −0.5 V vs Ag/AgNO_3_ (Figure 8a). Both samples exhibit two distinct semicircles that were well-fitted using an equivalent circuit containing a series resistance (*R*_*s*_) and two resistor-constant phase element (RQ) circuits in series. The *R*_*1*_ *Q*_*1*_ branch corresponds to the higher-frequency semicircle, with *R*_*1*_ representing the effective charge carrier transport resistance within the bulk CuFe_2_O_4_ material, while *Q*_*1*_ accounts for the capacitive contributions from the space charge region, Helmholtz layer, and chemical capacitance associated with restricted molecular diffusion.^52–55^ The *R*_*2*_ *Q*_*2*_ branch governs the lower-frequency carrier dynamics at the CuFe_2_O_4_-electrolyte interface, with *R*_*2*_ reflecting the resistance to heterogeneous charge transfer and *Q*_*2*_ representing electron accumulation in surface states.^56^ To account for the nonideal grain boundaries in polycrystalline CuFe_2_O_4_ films and the irregular surface morphology, constant phase elements (*Q*_*i*_) were used in the fitting rather than conventional capacitors. The nonideality of each constant phase element is captured by an exponent parameter *n*_*i*_ (0 < *n*_*i*_ < 1).

**Figure 8:**
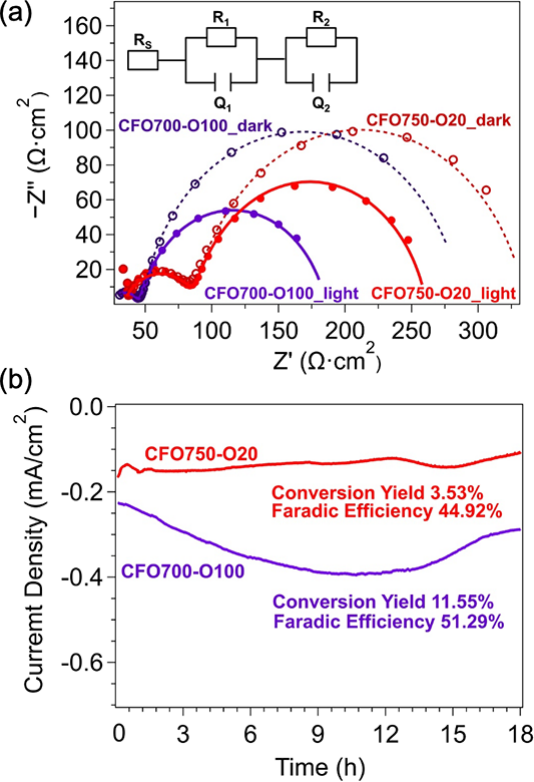
(a) PEIS Nyquist plots measured from CFO750-O20 and CFO700-O100 in 20 mL of 4:1 (v/v) acetonitrile/water mixture, with the addition of 0.1 M LiClO_4_, 0.4 mmol of 1,4-benzoquinone, and 0.4 mmol of benzaldehyde under an applied potential of −0.5 V vs Ag/AgNO_3_. The open (solid) circles represent the impedances at different modulation frequencies, and the dashed (solid) curves are the fitting results employing the equivalent circuit in the inset under dark (illuminated) conditions. (b) Comparison of chronoamperometry curves of CFO750-O20 and CFO700-O100 at −0.5 V vs Ag/AgNO_3_ for benzaldehyde hydrogenation.

The fitting results for the PEIS Nyquist plots are summarized in Table 1. Upon illumination, both photocathodes display a 30–40% reduction in *R*_*2*_, indicating more efficient charge transfer to the mediator due to surface photovoltage effects. Conversely, *R*_*1*_ remains largely unchanged between dark and illuminated conditions, consistent with transport-limited behavior set by bulk conductivity. Notably, CFO700-O100 shows a significantly lower *R*_*1*_ (22.3 Ω) than CFO750-O20 (53.7 Ω) under illumination, consistent with its higher hole concentration inferred from Mott–Schottky, XPS, UPS, and DFT analyses.

**Table 1:** Fitting Results for the PEIS Nyquist Plots of CuFe_2_O_4_ Photocathodes at −0.5 V vs. Ag/AgNO_3_

Sample	Condition	*R*_*s*_ (Ω)	*R*_*1*_ (Ω)	*Q*_*1*_ (μF)	*n*_*1*_	*R*_*2*_ (Ω)	*Q*_*2*_ (μF)	*n*_*2*_
CFO750-O20	Dark	33.8	54.1	1.72	0.719	248	98.1	0.863
CFO750-O20	Illuminated	33.4	53.7	1.05	0.756	174	97.9	0.851
CFO700-O100	Dark	24.4	23.2	1.47	0.660	241	180	0.876
CFO700-O100	Illuminated	23.5	22.3	0.989	0.675	140	156	0.836

CFO700-O100 also exhibits a larger interfacial capacitance *Q*_*2*_ (156 μF vs 97.9 μF), suggesting a higher density of surface states for accommodating photogenerated electrons. At the same time, a lower *R*_*2*_ compared to CFO750-O20 (140 Ω vs 174 Ω) under illumination implies more efficient 1,4-benzoquinone reduction relative to surface electron–hole recombination. Taken together, the enhanced photoelectrocatalytic performance arises from synergistic improvements in bulk charge transport and surface charge-transfer kinetics. These effects account for the substantially higher photocurrent observed for CFO700-O100 during PEC benzaldehyde reduction (Figure 8b). Although both films yield similar FEs (45–52%) for benzyl alcohol after 18 h, the higher photocurrent density of CFO700-O100 results in a more than 3-fold increase in conversion yield.

As discussed in the introduction section, PEC carbonyl reduction has only been previously demonstrated by Wang et al. using an α-Bi_2_O_3_ photocathode functionalized with Au clusters.^21^ In that work, α-Bi_2_O_3_ (*E*_*g*_ = 2.80 eV) exhibits minimal visible-light absorption, such that the Au clusters serve a dual role as both light absorbers and catalytic reduction sites. A photocurrent density of ∼0.35 mA/cm^2^ was reported at −0.15 V vs Ag/AgCl in phosphate buffer (pH 7) under 565 nm LED illumination, while the photocurrent response under simulated solar irradiation, the condition used for benzaldehyde reduction, was not reported. Using simulated solar illumination, ∼95% of 2 μmol benzaldehyde (0.1 mM) was consumed within 0.5 h at −0.15 V vs Ag/AgCl, corresponding to an ∼3.80 μmol/h conversion rate. In comparison, our CuFe_2_O_4_ photocathodes operated at a 200-fold higher substrate loading (0.4 mmol, 20 mM) and enabled direct identification and quantification of benzyl alcohol, thereby allowing determination of Faradaic efficiencies. At −0.50 V vs Ag/AgNO_3_ (−0.144 V vs Ag/AgCl), an 11.55% conversion yield was achieved over 18 h, corresponding to a benzyl alcohol production rate of 2.57 μmol/h.

Although this production rate is modestly lower than that reported for the Au-sensitized α-Bi_2_O_3_ system, the present approach relies a single-component, earth-abundant oxide with a narrower bandgap (∼1.7 eV), enabling more efficient solar light harvesting without noble-metal sensitizers. Moreover, stability in prior work was evaluated by repeated short (15 min) PEC cycles by monitoring benzaldehyde consumption, whereas the present study demonstrates sustained operation during a single, continuous 18-h photoelectrocatalysis experiment with direct product analysis.

From a practical perspective, the synthesis of Au nanoclusters requires expensive precursors and low reported yields, followed by additional losses during electrode loading, whereas CuFe_2_O_4_ can be synthesized at scale using standard sol–gel processing. Taken together, these results highlight complementary design strategies for PEC carbonyl reduction and underscore the potential for defect-engineered, earth-abundant oxide photocathodes for scalable organic transformations.

Compared to the relatively negative potentials (−1.0 to −1.3 V) typically required for electrocatalytic benzaldehyde reduction,^57,58^ our PEC approach operates within a more favorable potential window, reducing the required bias by approximately 1 V. Conventional electrocatalytic hydrogenation of carbonyl groups generally relies on initial substrate adsorption onto the electrode surface followed by proton-coupled electron transfer (PCET) steps,^13,59–61^ making the preferentially reduction of carbonyl groups over HER a persistent challenge. In contrast, the indirect photoelectrocatalysis strategy employed here decouples light absorption from interfacial reduction chemistry, enabling carbonyl reduction at less cathodic potentials while suppressing HER and mitigating self-reduction of the CuFe_2_O_4_ photocathode. These results broaden the scope of photoelectrocatalysis for selective organic reduction reactions. Looking forward, further advances in photoelectrochemical organic transformations may benefit from combining defect engineering with advanced materials-design strategies developed in heterogeneous photocatalysis, such as nanostructured hybrids and bioinspired architectures.^62,63^

## Conclusions

This study establishes a defect-engineering and redox-mediated strategy to enhance the photoelectrocatalytic performance of CuFe_2_O_4_ photocathodes for the reduction of benzaldehyde. By tuning the annealing atmosphere, CFO700-O100 achieved substantial passivation of oxygen vacancies, as evidenced by LSV, Mott–Schottky, XPS, UPS, and defect-formation-energy calculations. This passivation increased the hole concentration and reduced both charge transport and interfacial charge transfer resistances, resulting in markedly improved photocurrent densities relative to CFO750-O20. Stabilization of the photocathode was further achieved through the incorporation of 1,4-benzoquinone as a redox mediator, which suppressed surface photocorrosion and limited competing hydrogen evolution. Under these optimized conditions, CFO700-O100 delivered over three times the benzaldehyde-to-benzyl-alcohol conversion rate of CFO750-O20, despite similar Faradaic efficiencies. Overall, this study demonstrates that combining defect control with redox-mediated charge management provides an effective route to improving both stability and catalytic performance of metal-oxide photocathodes. These insights highlight a broader potential for sustainable photoelectrocatalytic organic transformation. Future efforts will focus on closing the mediator cycle and independently quantifying competing reactions to further improve Faradaic efficiency.

## Supplementary Material



## Notes

Plane-view and cross-sectional SEM images of CuFe_2_O_4_ films; GIXRD patterns; Mott–Schottky plots measured between 100–5000 Hz; Cu 2*p* and Fe 2*p* XPS spectra; Cu LMM Auger spectra; chronoamperometry curves; UV–vis absorption spectra and Tauc plots of CuFe_2_O_4_ films; GIXRD, SEM, EDX, and UV–vis absorption spectra of electrolyte before and after PEC benzaldehyde reduction reaction; additional DFT+U calculation details (PDF)
