# Supplementary material for: Unraveling Defect-Dependent Conductivity-Type Switching in CuFe2O4 for Enhanced Photoelectrocatalytic Reduction of Benzaldehyde
Source: ACS Appl Mater Interfaces. 2026 Feb 19;18(8):12759–69. doi: 10.1021/acsami.5c24965 (PMC12964343; doi:10.1021/acsami.5c24965)
Supplement: Supplementary file 1 [file am5c24965_si_001.pdf]

<u>Supporting Information</u>

Unraveling Defect-Dependent Conductivity-Type Switching in $CuFe_2O_4$ for Enhanced Photoelectrocatalytic Reduction of Benzaldehyde

Yen-Chun Huang,[1] Manoj Kumar Mohanta,[1] Jun-Lin Fong[1], Abdul M. Reyes,[3,4] Sebastian E. Reyes-Lillo,[3] Chang-Ming Jiang[1,2,*]

[1]*Department of Chemistry, National Taiwan University, 106319 Taipei, Taiwan*

[2]*Center for Emerging Materials and Advanced Devices, National Taiwan University, 106319 Taipei, Taiwan*

[3]*Departamento de Física y Astronomía, Facultad de Ciencias Exactas, Universidad Andres Bello, 837-0136 Santiago, Chile*

[4]*SPN Tumaco, Universidad Nacional de Colombia, Kilómetro 30-31, Vía Nacional Tumaco, Colombia*

*Corresponding Author: cmjiang@ntu.edu.tw

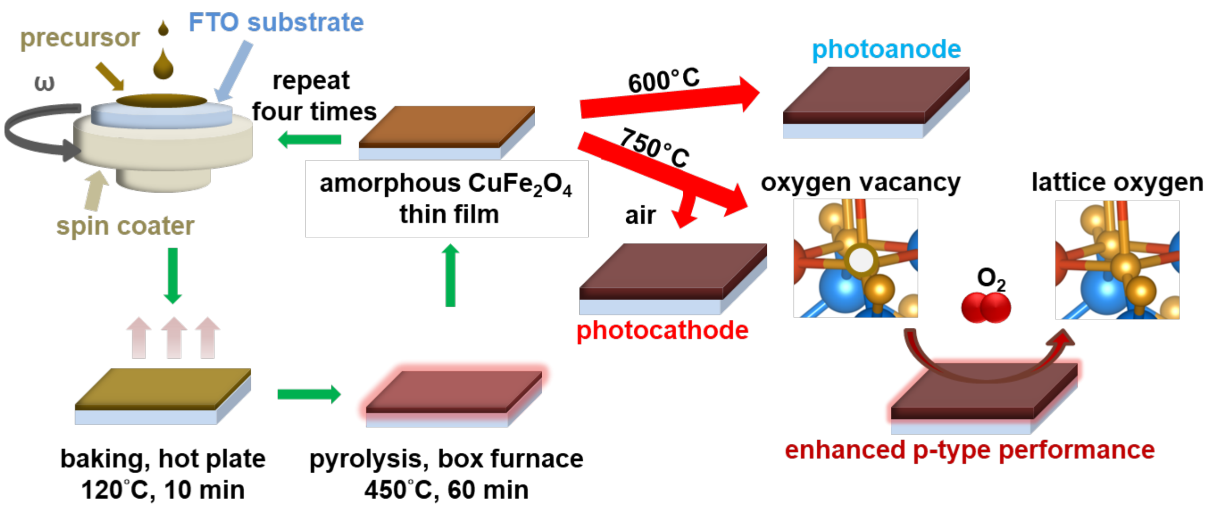

Figure S1. The sol-gel spin-coating procedure for depositing $CuFe_2O_4$ films on FTO glass substrates.

Characterization of $CuFe_2O_4$ Films

Grazing incidence X-ray diffraction (GIXRD) measurements were performed on a Rigaku SmartLab SE diffractometer equipped with a Cu anode (λ = 1.54178 Å), using a 0.5° incident angle over a 2θ range of 28–48°. Raman scattering spectra were obtained using a UniNano UNIDRON-A spectrometer with a 633 nm excitation source. The surface morphology of the $CuFe_2O_4$ films was examined using a JSM-7600F (JEOL) field emission scanning electron microscope in secondary electron detection mode.

Ultraviolet-visible (UV-Vis) transmission spectra were collected over the 400–750 nm wavelength range using a Jasco V-770 spectrometer. The optical bandgaps of the $CuFe_2O_4$ thin films were determined using Tauc plots, where the absorption coefficient (α), photon energy ($h\nu$), and bandgap energy (E_g) were fitted to the equation $(\alpha h\nu)^{1/n} = B(h\nu - E_g)$, assuming n = 2 for indirectly allowed interband transitions.

X-ray photoelectron spectroscopy (XPS) was carried out using an Ulvac PHI 5000 Versa Probe system with a monochromatic Al K_α source ($h\nu$ = 1486.6 eV); ultraviolet photoelectron spectroscopy (UPS) was conducted on the same apparatus using He-I radiation. All core-level binding energies were referenced to the adventitious C 1s peak at 284.8 eV. XPS and UPS data were analyzed using CasaXPS software.

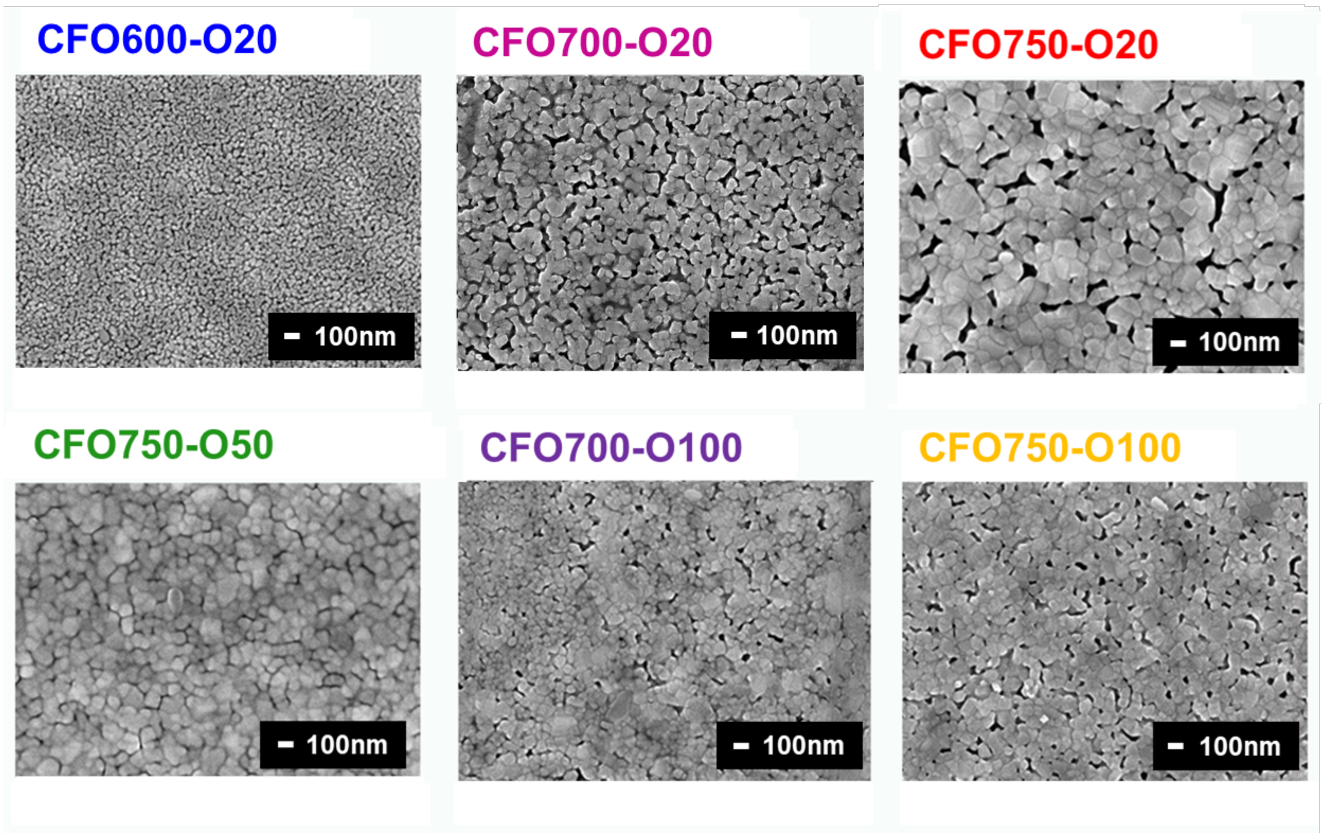

Figure S2. Plane-view scanning electron microscopy (SEM) images of $CuFe_2O_4$ thin films synthesized with different annealing temperatures and atmospheres.

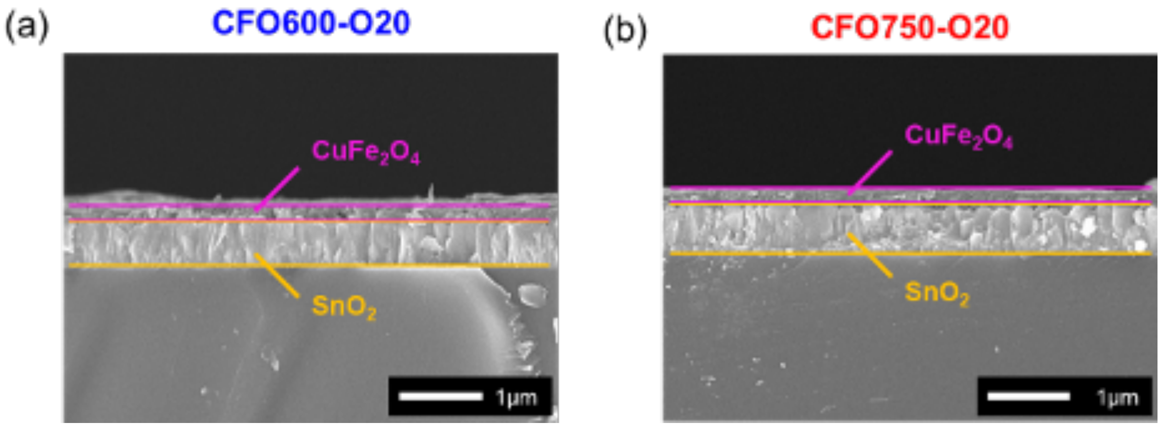

Figure S3. Cross-sectional SEM images of (a) CFO600-O20 and (b) CFO750-O20.

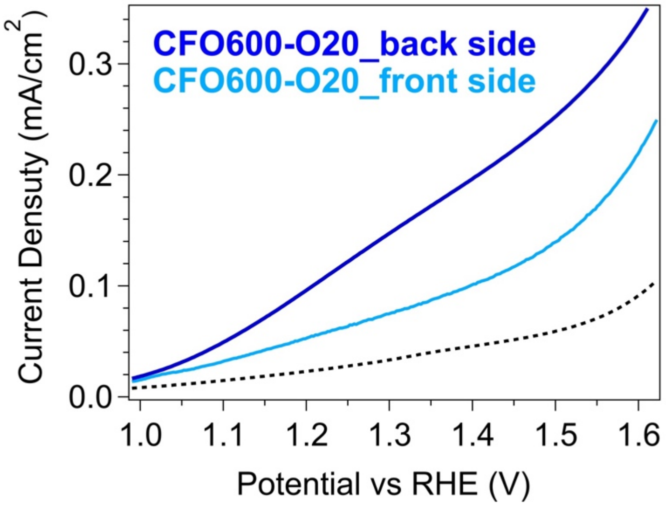

Figure S4. Linear scan voltammetry (LSV) plots measured from CFO600-20 using front-side and back-side AM 1.5G illuminations at 100 mW/cm^2 intensity in 1 M NaOH with 0.2 M Na_2SO_3 added as a hole scavenger. The dashed line was acquired in dark conditions.

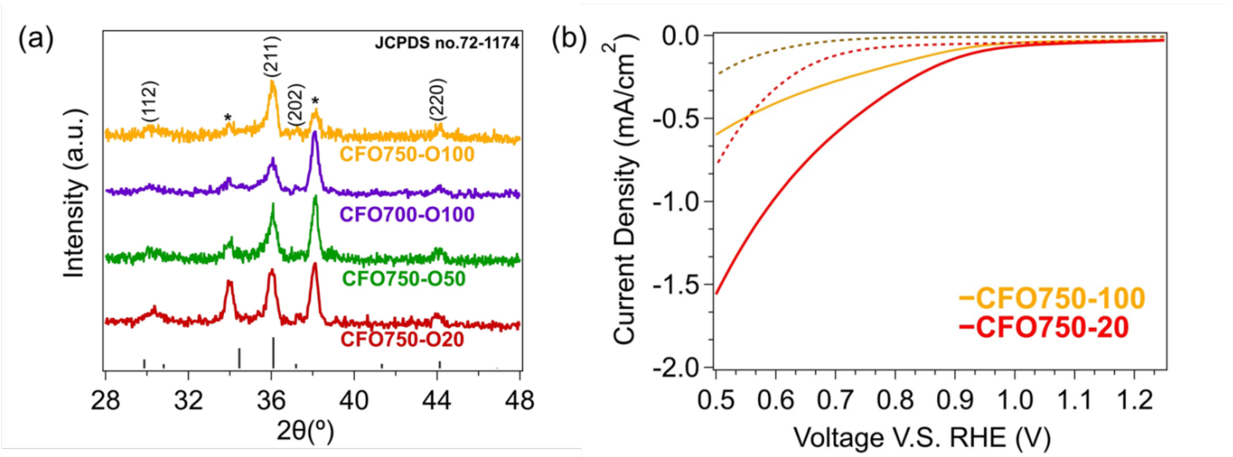

Figure S5. (a) Grazing incidence X-ray diffraction patterns of $CuFe_2O_4$ films synthesized with different annealing conditions. The asterisks indicate the diffraction pattern of FTO. (b) LSV plots measured from CFO750-O20 and CFO750-O100 in 1 M NaOH and 0.2 M $Na_2S_2O_8$.

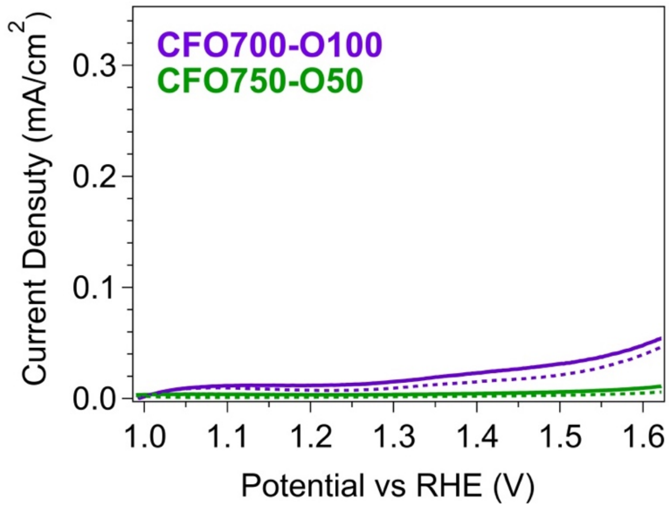

Figure S6. LSV plots measured from CFO750-50 and CFO700-100 in 1 M NaOH with 0.2 M Na_2SO_3. The dashed lines were acquired in dark conditions, while the solid lines were recorded under back-side illumination of AM 1.5 G simulated solar light at 100 mW/cm^2 intensity.

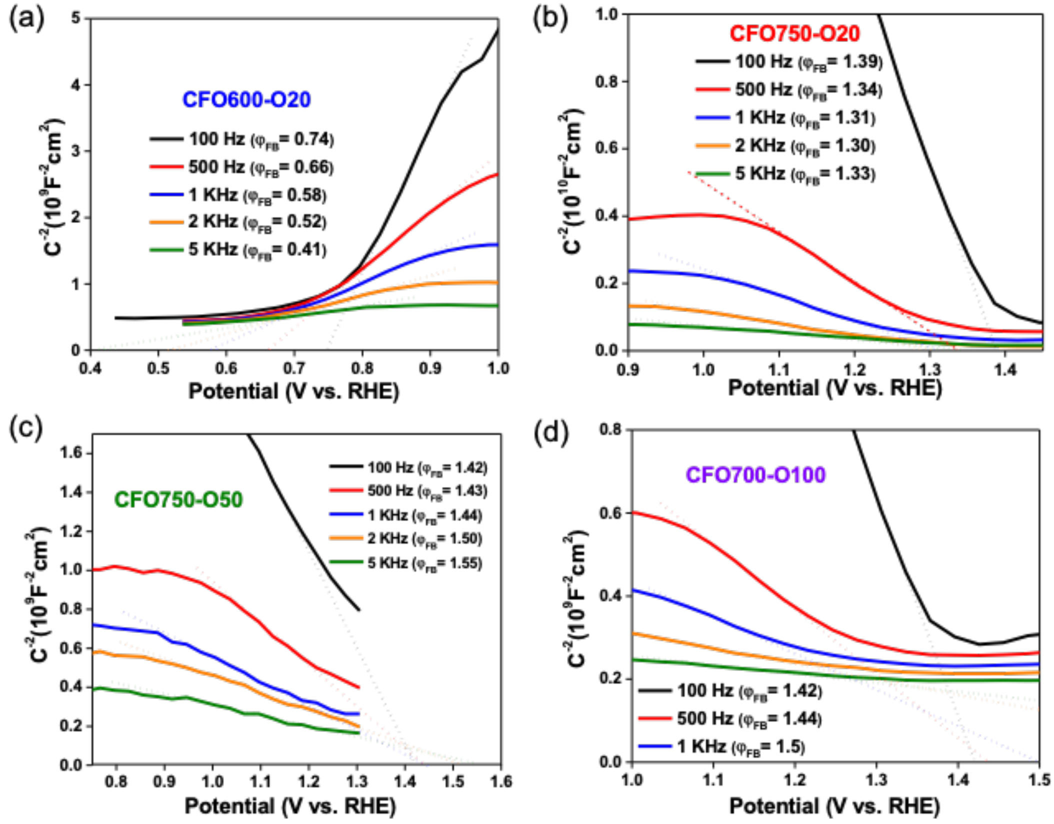

Figure S7. Mott-Schottky plots measured from (a) CFO600-O20, (b) CFO750-O20, (c) CFO750-O50, and (d) CFO700-O100 using different modulation frequencies.

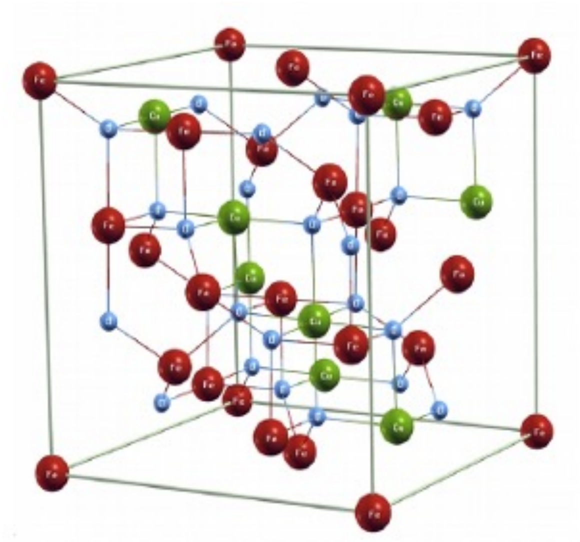

Figure S8. The structural and magnetic optimization of the 52-atom pristine $CuFe_2O_4$ 2×2×2 supercell employed to construct the 448-atom supercell, which was utilized to calculate the formation energies and transition levels of point defects.

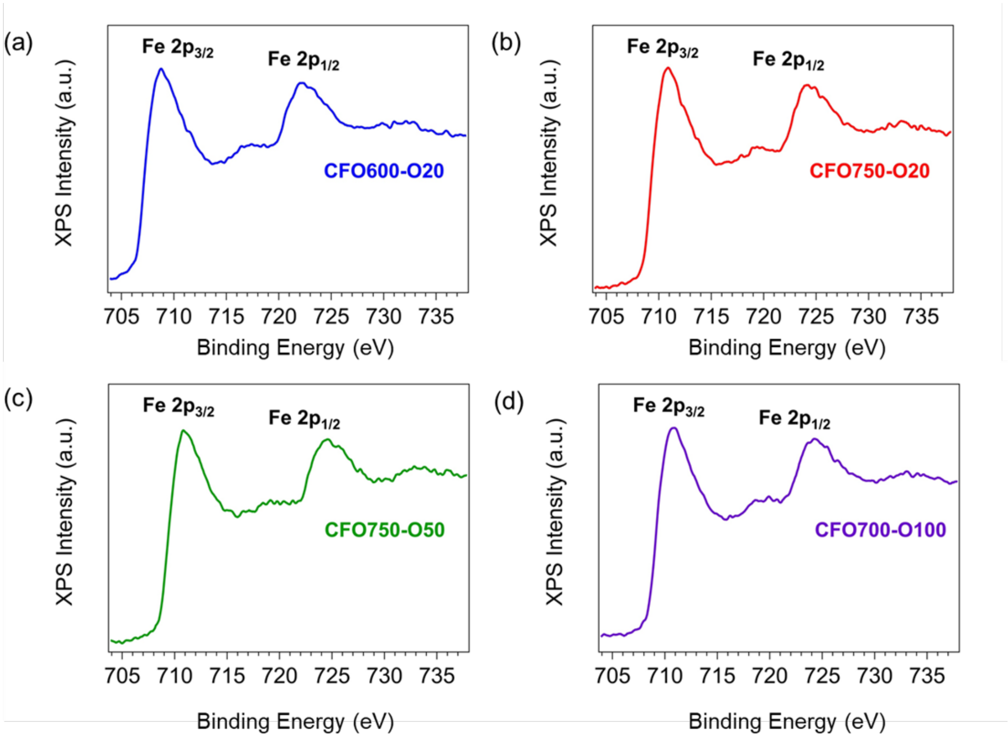

Figure S9. High-resolution Fe 2*p* X-ray photoemission spectra of (a) CFO600-O20, (b) CFO750-O20, (c) CFO750-O50, and (d) CFO700-O100.

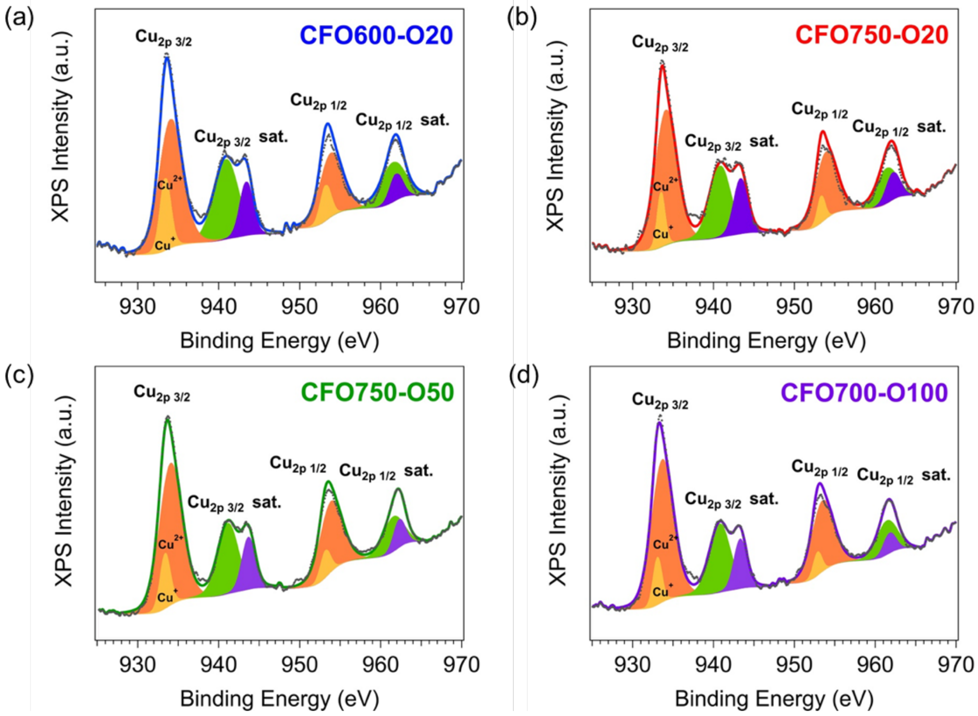

Figure S10. High-resolution Cu 2*p* X-ray photoemission spectra of (a) CFO600-O20, (b) CFO750-O20, (c) CFO750-O50, and (d) CFO700-O100. Experimental results are shown as dots, while the fitted envelopes are represented as solid lines.

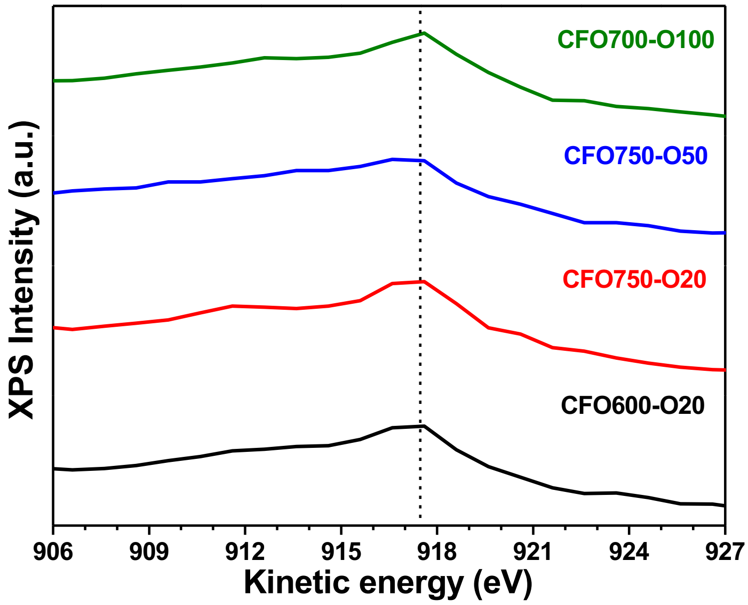

Figure S11. Cu LMM Auger spectra of CFO600-O20, CFO750-O20, CFO750-O50, and CFO700-O100.

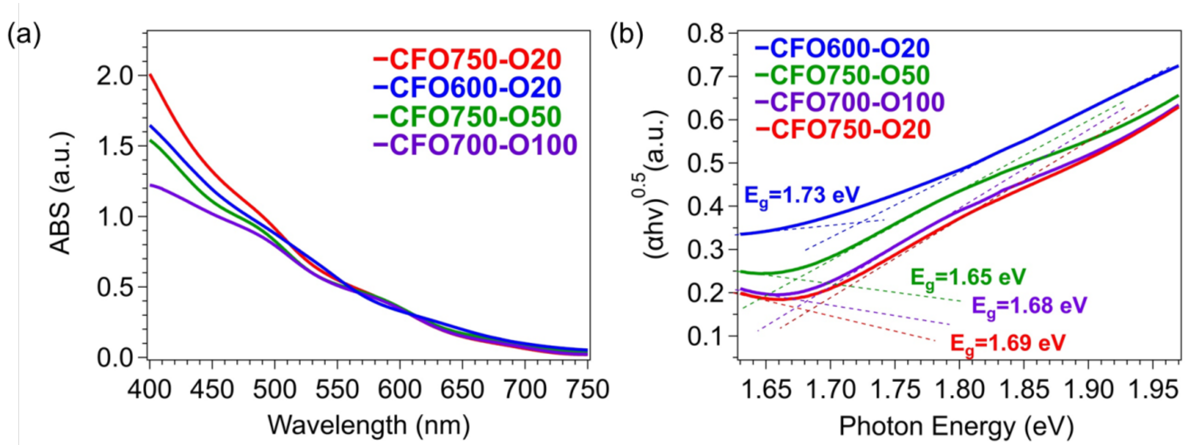

Figure S12. (a) Absorption spectra of different $CuFe_2O_4$ photoelectrodes. (b) Tauc analysis results for indirectly allowed interband optical transitions.

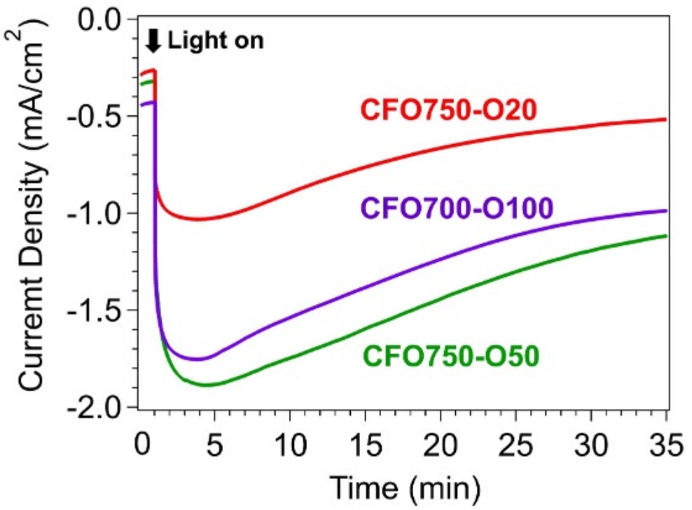

Figure S13. Chronoamperometry curves measured from three $CuFe_2O_4$ photocathodes – CFO750-O20, CFO750-O50, and CFO700-O100 – at 0.50 V vs. RHE under AM 1.5G illumination at 1 sun intensity. The aqueous electrolyte consisted of 1 M NaOH and 0.2 M $Na_2S_2O_8$.

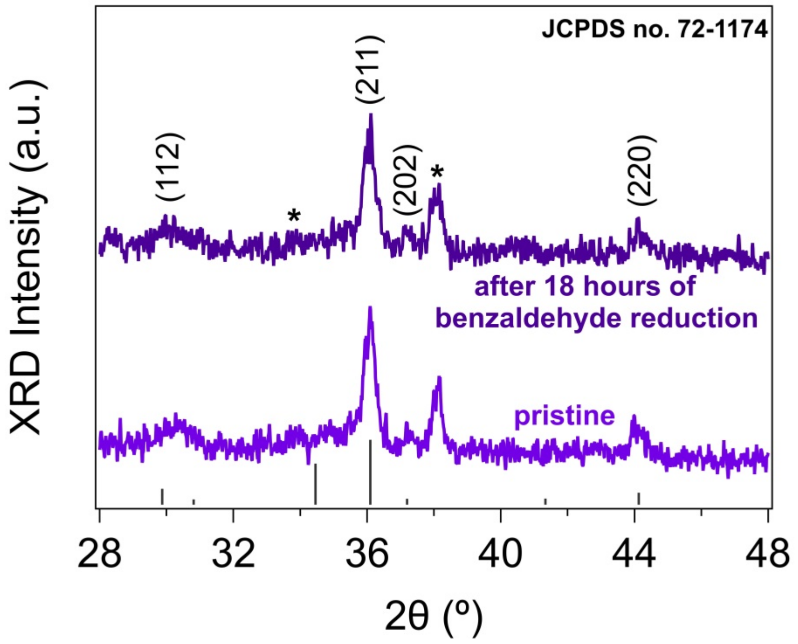

Figure S14. Comparison of the grazing incidence X-ray diffraction patterns measured from CFO700-O100 before and after 18 hours of photoelectrocatalytic benzaldehyde reduction reaction. The asterisks indicate the diffraction peaks of SnO_2 in the FTO glass substrates.

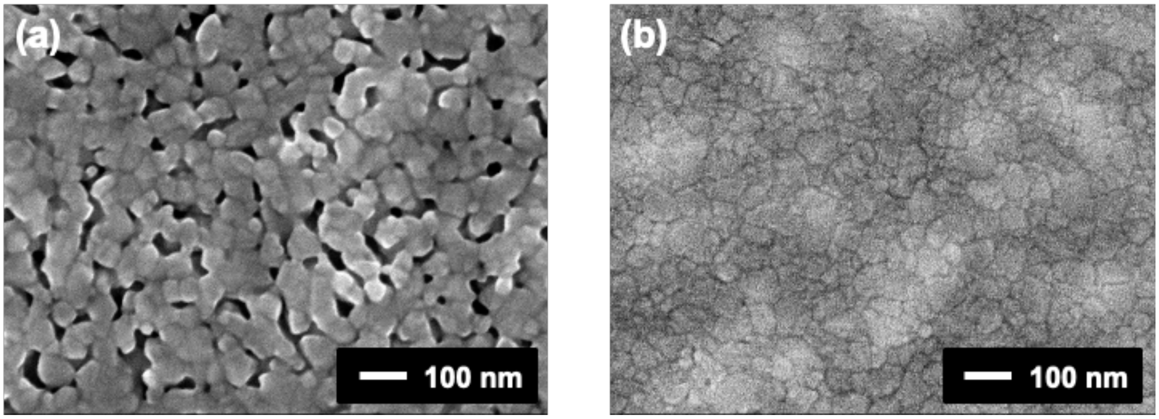

Figure S15. Plane-view SEM images of CFO700-O100 photocathodes (a) before and (b) after 18 hours of photoelectrocatalytic benzaldehyde reduction reaction.

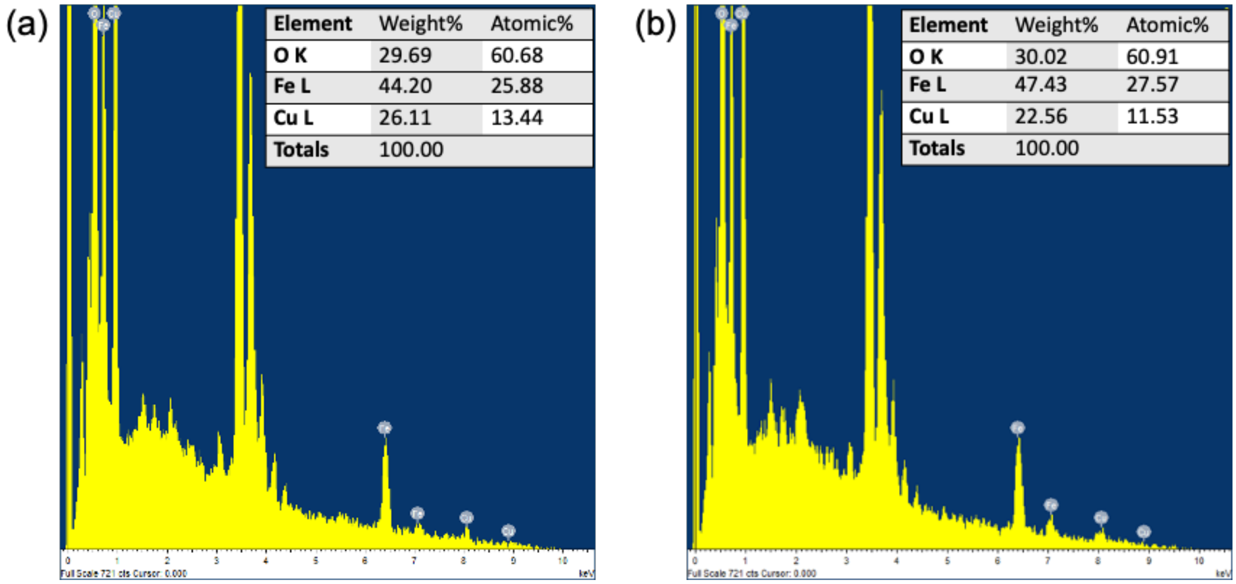

Figure S16. Energy-dispersive X-ray (EDX) spectra of CFO700-O100 photocathodes (a) before and (b) after 18 hours of photoelectrocatalytic benzaldehyde reduction reaction.

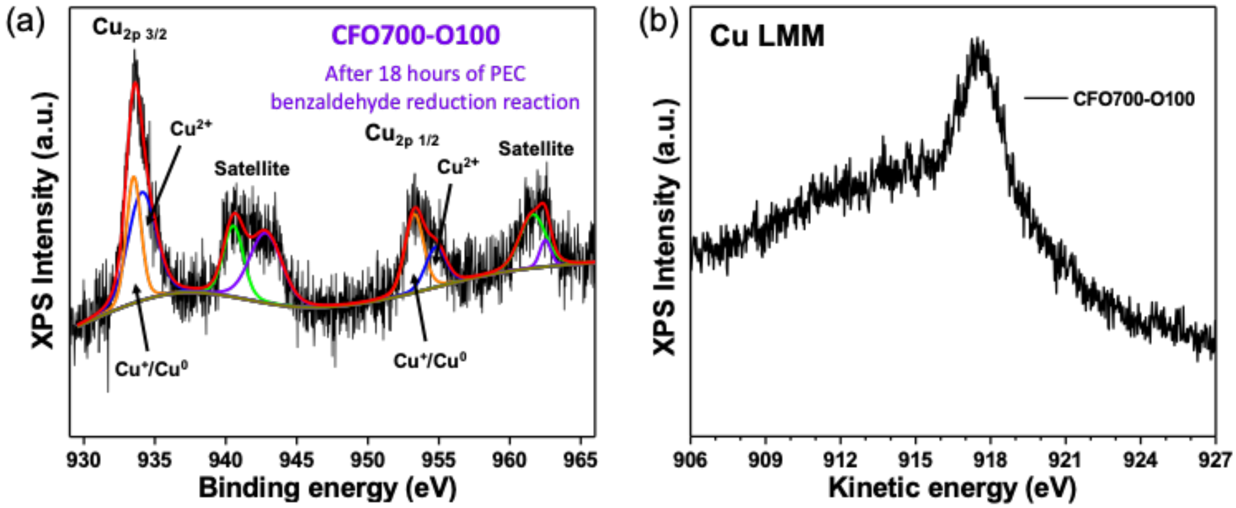

Figure S17. (a) Cu 2*p* XPS and (b) Cu LMM Auger spectra measured from CFO700-O100 after 18 hours of continuous photoelectrocatalytic benzaldehyde reduction.

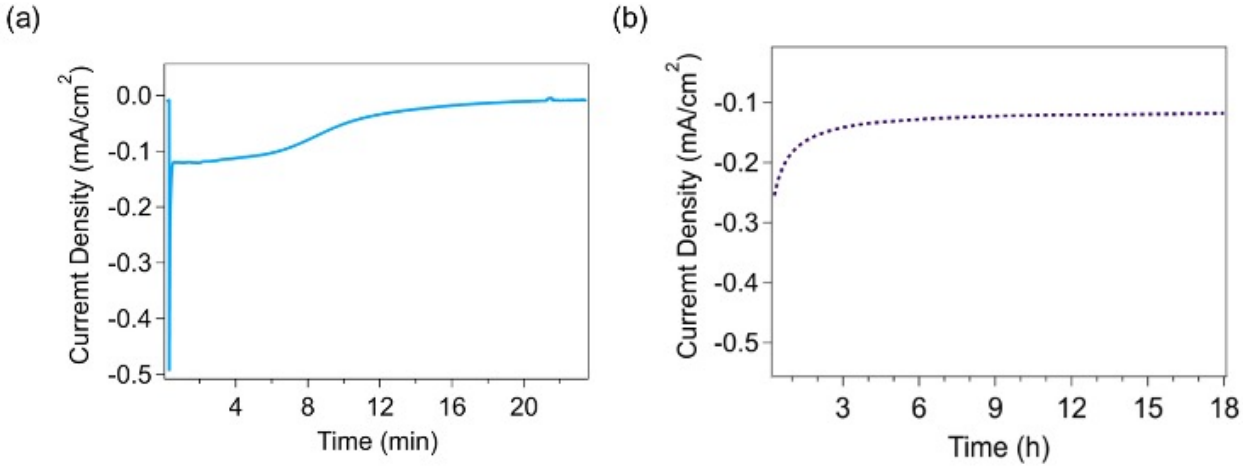

Figure S18. Chronoamperometry curves measured from CFO700-O100 for PEC benzaldehyde hydrogenation at –0.50 V vs. $Ag/AgNO_3$ (a) without 1,4-benzoquinone and (b) without AM 1.5 G illumination.

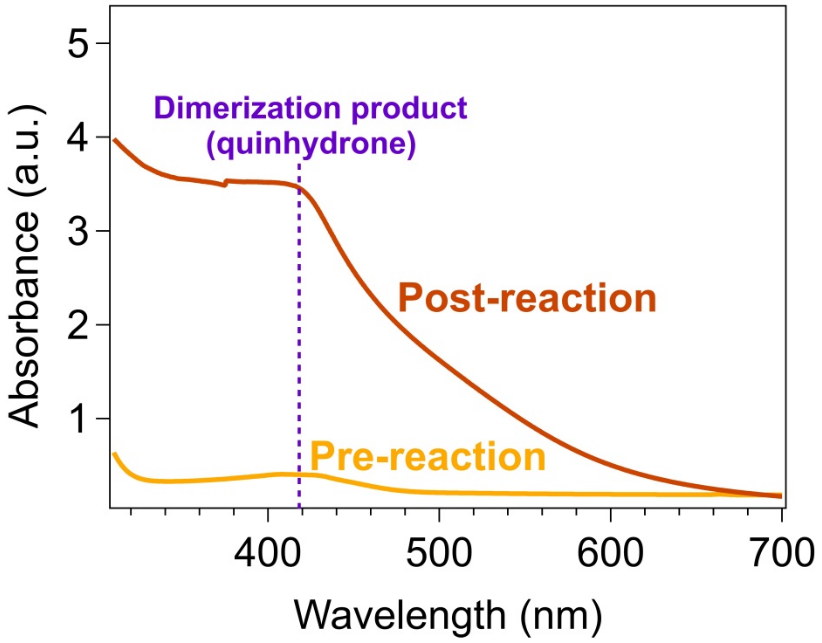

Figure S19. UV-vis absorption spectra of the mixed acetonitrile/water electrolyte solution before and after 18 hours of photoelectrocatalytic benzaldehyde reduction reaction.

Computation Details

Spin-polarized density functional theory (DFT) calculations were performed using the VASP software package.[43] Structural and magnetic optimizations were conducted at the DFT+U level using the generalized gradient approximation (GGA) plus the Hubbard U correction method.[44,45] Our calculations used a plane-wave cutoff energy of 400 eV and 4×4×4 k-points grids. The effective on-site correlation Hubbard U value for Cu and Fe were set to 5.0 eV and 5.3 eV, respectively.[46,47] The electron-ion interaction was described using the projector augmented wave (PAW) pseudopotentials,[48,49] with 11, 8, and 6 electron valence electrons for Cu ($3d^{10}4s^1$), Fe ($3d^6 4s^2$), and O ($2s^2 2p^4$), respectively.

The formation energy $H(X^q)$ of a defect X with charge q was computed as a function of the Fermi level E_F using the expression:[50,51]

$$H(X^q) = E\,[X^q] - E\,[bulk] + \Sigma\, n_i\, \mu_i + q(E_F + E_{VB}) + \Delta E_{size}$$

where $E[X^q]$ and $E[bulk]$ represent the total energy of the supercell containing the defect and the pristine bulk 2×2×2 supercell, respectively. The parameters n_i and μ_i denote the number and chemical potential of the ions introduced to ($n_i < 0$) or removed from ($n_i > 0$) the supercell. E_{VB} is the valence band maximum of the pristine supercell, and q is the number of electrons added ($q < 0$) or removed ($q > 0$) to the supercell. The computed chemical potentials for Cu, Fe, and O are listed in **Tables S1-S2**.

The simulation of Cu and O vacancies was conducted in a 448-atom supercell. Formation energies and transition levels were obtained from total energy calculations employing DFT+U. Supercell calculations were performed with a plane-wave energy cutoff of 400 eV and Γ-centered single-point grids. The optimized supercell lattice parameter, L = 16.5 Å, is sufficiently large to avoid interaction between defects in neighboring supercell images. However, due to the long-range nature of the Coulomb interaction, a size correction, ΔE_{size}, is necessary for charged defects. Here, the size correction is chosen as the sum of a potential alignment correction and a simplified form of the Makov-Payne correction, as defined by Lany and Zunger:[52]

$$\Delta E_{size} = q\Delta V + \alpha q^2/L.$$

where ΔV represents the average potential energy difference computed at a considerable distance from the defect in the supercell and in the pristine bulk supercell. In this work, the coefficient α is approximated by a Madelung term (0.4 eV Å) to account for the size effect and is applied to the charged defect states.[53]

The stability range of $CuFe_2O_4$ relative to the competing phases, such as CuO and Fe_2O_3, is shown in **Table S1**. According to the thermodynamic equilibrium conditions, the sum of the chemical potentials of Cu, Fe, and O atoms and the enthalpy of formation of $CuFe_2O_4$ must be equal to maintain the equilibrium and is given as:

$$\Delta H(CuFe_2O_4) = (\mu_{Cu} + 2\,\mu_{Fe} + 4\,\mu_{O}) - (\mu_{elemCu} + 2\,\mu_{elemFe} + 4\,\mu_{elemO})$$

$$= \Delta\mu_{Cu} + 2\,\Delta\mu_{Fe} + 4\,\Delta\mu_{O}.$$

To avoid the formation of competing phases, the following conditions must be satisfied simultaneously:

$$a\Delta\mu_x + b\Delta\mu_y + c\Delta\mu_z \leq \Delta H(X_aY_bZ_c),$$

where x, y, and z correspond to Cu, Fe, and O, respectively, and $\Delta H(X_aY_bZ_c)$ are the enthalpies of formations of the competing phases (**Table S2**).

Table S1. Enthalpies of formation for the relevant Cu-Fe-O crystalline phases, calculated using PBE+U functionals.

**Competing phases**	**Formation energies (eV)**
$CuFe_2O_4$	–11.06
CuO	–1.18
Cu_2O	–0.74
Cu_4O_3	–4.40
Fe_2O_3	–9.48
Fe_3O_4	–13.25
$FeCuO_2$	–5.76

Table S2. Chemical potentials for Cu, Fe, and O_2 obtained with PBE+U functionals.

Element	Elemental chemical potential, μ_{elem} (eV)	$\Delta\mu$	
		O-rich (eV)	O-poor (eV)
Cu	–2.06	–0.10	–0.60
Fe	–4.42	–4.00	–3.40
O_2	–5.44	–0.50	–0.90

Table S3. Configurations considered to find the lowest energy ground state of the $CuFe_2O_4$ system. Each structure was optimized using the PBE+U functional.

Alloys	Total energies (eV)
Configuration 1 (FM)	–330.99
Configuration 2 (FM)	–330.90
Configuration 3 (FM)	–330.91
Configuration 4 (FM)	–331.34
Configuration 5 (FM)	–331.91
Configuration 6 (FM)	–331.29
Configuration 7 (FM)	–331.39
Configuration 8 (AFM)	–333.83
Configuration 9 (AFM)	–333.78
Configuration 10 (AFM)	–333.56
